# Experimental Investigation on the Impact of Graphite Electrodes Grain Size on Technological Parameters and Surface Texture of Hastelloy C-22 after Electrical Discharge Machining with Negative Polarity

**DOI:** 10.3390/ma17102257

**Published:** 2024-05-10

**Authors:** Rafał Nowicki, Dorota Oniszczuk-Świercz, Rafał Świercz

**Affiliations:** Institute of Manufacturing Technology, Faculty of Mechanical and Industrial Technology, Warsaw University of Technology, Narbutta 85, 02-524 Warsaw, Poland; dorota.swiercz@pw.edu.pl

**Keywords:** EDM, graphite electrodes, tool wear rate, material removal rate, surface roughness

## Abstract

Electrical discharge machining (EDM) is a rapidly evolving method in modern industry that manufactures highly complex components. The physical properties of a tool electrode material are significant factors in determining the effectiveness of the process, as well as the characteristics of the machined surfaces. The current trend of implementing graphite tool electrodes in manufacturing processes is observed. Innovative material engineering solutions enable graphite production with miniaturized grain size. However, the correlation between the graphite electrode grain size and the mechanism of the process removal in the EDM is a challenge for its widespread implementation in the industry. This research introduces a new method to evaluate the impact of the graphite electrode grain size and machining parameters on the material removal effectiveness, relative tool wear rate, and surface roughness (*Ra*) of Hastelloy C-22 following EDM with negative polarity. The study utilized new graphite materials with a grain size of 1 µm (POCO AF-5) and 10 µm (POCO EDM-180). An assessment of the impact of the EDM process parameters on the technological parameters and the development of the surface roughness was carried out. Electrical discharge machining with fine-grained graphite electrodes increases process efficiency and reduces tool wear. Graphite grains detached from the tool electrode affect the stability of electrical discharges and the efficiency of the process. Based on the experimental results, mathematical models were developed, enabling the prediction of machining effects to advance state-of-the-art manufacturing processes. The obtained mathematical models can be implemented in modern industrial EDM machines as guidelines for selecting adequate machining parameters depending on the desired process efficiency, tool wear rate, and surface roughness for advanced materials.

## 1. Introduction

**Figure 1:**
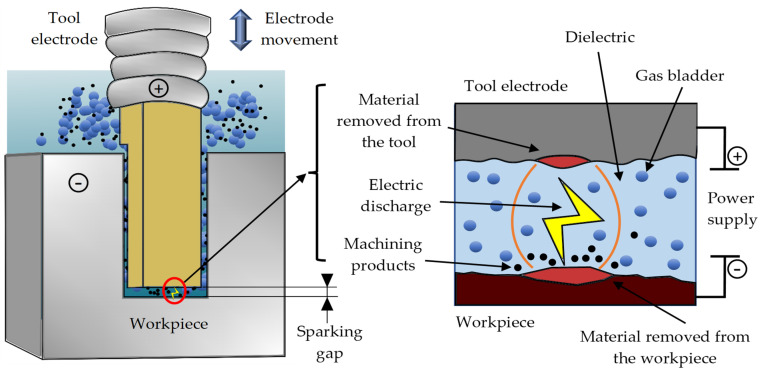
EDM scheme.

Electrical discharge machining (EDM) is an unconventional method of treatment in which material is removed as a consequence of electrical discharges occurring in a liquid or gaseous dielectric medium between two separated electrodes (Figure 1) [1,2,3,4,5]. The electrodes are connected to the power supply. A workpiece is one of the electrodes. The second electrode is the tool electrode that manufactures the shape during the machining of the workpiece. Depending on current pulse waveforms, the values of their parameters, and the material of the electrodes, the electro-erosion process can be carried out with positive or negative polarization. In negative polarity, the tool electrode is connected to the negative pole of the current, so it is the cathode, and the workpiece is the anode. In the opposite configuration, the polarity is positive [6,7]. The choice of polarity is mainly determined by the higher efficiency of material removal from the workpiece [8,9,10,11,12,13,14]. The phenomenon of material removal in the EDM process is complex, and it relies on the concept of thermoelectric erosion caused by electrical sparks between two conductive materials. The voltage between the cathode and the anode generates an inhomogeneous and time-varying electric field in the sparking gap [15,16]. With a sufficiently high electric field intensity (10^7^–10^8^ V/m), electrons are emitted from the cathode surface. When electrons are sped up in an electric field, they collide with the atoms in the dielectric medium, leading to avalanche impact ionization. This forms a thin plasma channel packed with electrons and positively charged ions released from the anodic side [17,18]. The kinetic energy of the electrons colliding with the anode surface and the ions colliding with the cathode surface is converted into thermal energy. The generated heat also contributes to the evaporation of the dielectric and creates high-pressure waves that intensify the removal of the molten material from the sparking gap [19,20]. The electric current flowing through the plasma channel during the spark discharge causes local heating of a particular volume of the electrode material to the melting point and then to the boiling point. The melted volume of the electrode material begins to evaporate, resulting in the generation of spherical recesses called craters. A gas bubble is formed around the plasma channel, which undergoes implosional breakdown after the end of the electric discharge. This promotes the removal of the melted material [21,22,23,24,25,26,27,28,29].

The material removal phenomenon in the EDM process is based mainly on electricity, which is converted into thermal energy due to electrical discharges. Intense heat induction into the material leads to melting, evaporation, and sublimation in local areas of the workpiece and the tool electrode [30,31]. The surface texture after machining consists of overlapping spherical craters created by a single electrical discharge. Local microstructure changes occur in the surface texture due to the impact of local thermal processes [32,33]. The shape and depth of the craters directly affect the surface roughness of the workpiece and strictly depend on the technological parameters of EDM, which determine the amount of material removed from the crater during a single electric discharge. The main factors that affect the surface roughness of the workpiece and the material removal efficiency are current intensity *I*_c_, discharge voltage *U*_c_, electrical discharge time *t*_on_, time interval *t*_off_, the physical properties of the tool and the workpiece, and the type of liquid or gaseous dielectric [34,35,36,37,38].

During electrical discharge machining, the tool electrode and the workpiece wear simultaneously. The extent of wear of the tool electrode can be minimized by an adequate selection of technological parameters of the process and the tool material with appropriate thermo-physical properties [39]. The principal properties of the tool electrode material that affect the EDM process are electrical and thermal conductivity, melting/sublimation point, density, and thermal expansion [40]. During machining, an electric current flows through the external circuit and then through the sparking gap in the form of electrons. The higher electrical conductivity of the tool electrode emits more electrons in the plasma channel, which can melt more material from the workpiece [41]. High thermal conductivity prevents high-temperature generation on the active surface of the tool and reduces its wear [42]. The high melting point of the tool electrode material prevents erosive wear caused by the elevated temperature of the plasma channel generated in the interelectrode gap. The density of a material is a measure of the packing of atoms in a particular volume. Higher-density materials wear less. Thermal expansion affects the accuracy of machining and the conditions for removing machining products from the interelectrode gap. In EDM, tool electrodes with low thermal expansion are preferred [43,44,45,46,47,48]. For years, copper electrodes have been the most used materials in the EDM process due to their high thermal and electrical conductivity. The disadvantage of copper electrodes is their high thermal expansion, which may worsen the conditions for rinsing machining products from the sparking gap and affect the accuracy of the manufactured shape. Copper also has a low melting point, which increases tool wear rate under certain machining conditions. Another disadvantage of copper is its ductility, which makes machining and deburring difficult. Adding tellurium to copper improves its machinability but lowers the process performance and increases the tool wear rate compared to pure copper [40,49,50].

Nowadays, graphite electrodes are increasingly being implemented in manufacturing processes due to their good machinability and the low purchase cost of raw materials. Graphite is the dominant material used for tool electrodes in the USA, and approximately 95% of the electrodes are made of this material. Mercer [51] pointed out that over 70% of the global market currently uses graphite for tool electrodes in EDM. Graphite materials include natural and artificial graphite, which is widely used for developing energy storage in various modern equipment. In addition, natural graphite can be divided into vein (lump), graphite, crystalline (flake), and aphanitic (microcrystalline, amorphous) graphite in terms of crystal particle size [52,53]. Graphite used in electrical discharge machining is classified according to grain size. Grain size and porosity determine the basic mechanical and thermophysical properties of graphite and thus have a different impact on the physics of the material removal phenomenon in EDM. Sonker [54] investigated the performance of the EDM process while machining high-carbon chromium die-steel using copper and graphite electrodes. The graphite electrode obtained the highest process efficiency compared to the copper electrode. The copper electrode provided a lower surface roughness of the workpiece. Lamba [55] conducted experiments on machining EN31 steel with copper and graphite electrodes in an abrasive mixed rotary tool EDM. The graphite electrode provided a higher process performance and lower surface roughness with the same machining conditions. Torres et al. [56] examined the impact of POCO EDM-1 graphite tool polarity on the technological indicators and the surface integrity of Inconel 600. The results indicate that higher material removal efficiency was obtained during machining with negative polarity. Lower surface roughness values and low wear of the tool electrode were obtained during EDM with positive polarity. It was found that negative polarity is recommended for roughing and positive polarity for finishing. Klocke et al. [57] investigated the effect of five different graphite grades on process efficiency and tool wear rate. The current intensity was the dominant parameter influencing the material removal efficiency, while the electric discharge time determined the wear of the tool. The researchers pointed out that the primary physical property of the graphite electrode affecting the performance of the EDM process is electrical conductivity. Furthermore, the most important factors influencing the tool wear mechanism are the grain size and the electrical conductivity of the graphite electrode. Aas et al. [58] showed that during the machining of turbine vanes made of nickel alloy, a coarse-grained graphite electrode obtained a significantly higher material removal rate compared to a fine-grained graphite electrode. However, the graphite electrode with a smaller grain achieved lower tool wear. Amorim et al. [59] reported that an increase in the graphite grain sizes increases the process performance by detaching larger particles from the tool due to the spalling effect, which then collide with the melted volume of material on the workpiece and remove it. However, using graphite electrodes with huge grains reduces the process efficiency due to the high contamination of debris and graphite particles in the interelectrode gap. Nowicki et al. [42] noticed that, in general, graphite electrodes with larger grains have higher electrical conductivity. During EDM of Hastelloy C-22 with positive polarization, the tool electrode made of graphite with a grain size of 1 µm achieved a lower material removal rate than the graphite tool electrode with a grain size of 10 µm. This is mainly due to the higher electrical conductivity of the coarse graphite. The fine-grained graphite electrode achieved a lower tool wear rate due to higher effective density.

The originality of this article is the comprehensive analysis of the effect of the grain sizes in the graphite tool electrode and process parameters of the electrical discharges in conditions of negative polarity machining on the technological parameters (process performance and tool wear) and surface integrity of the workpiece. Current scientific investigation indicates that the wear mechanism of graphite electrodes is caused by two phenomena: sublimation and mechanical detachment of graphite grains from the tool due to the impact of high-energy electric discharges. The complexity of the physics of material removal in the gap makes it difficult to predict the effect of the tool electrode properties and the process parameters on technological indicators such as wear of the graphite tool electrode, material removal rate, and surface texture condition. Therefore, studying new, difficult-to-machine materials allows for determining the relations between the technological indicators and the process parameters. Based on these dependencies, an adequate technology is built that can be adopted in modern electro-erosion machines.

## 2. Materials and Methods

The objective of the experimental study was to analyze how the size of the grains in POCO graphite electrodes and electrical variables affect the relative tool wear rate (TWR), material removal rate (MRR), and surface roughness (*Ra*) of Hastelloy C-22 following EDM with negative polarity. Hastelloy C-22 was chosen due to its extensive use in the chemical, petrochemical, and pharmaceutical industries [60]. Thanks to its high chromium, molybdenum, and tungsten content, the alloy provides excellent oxidation resistance, as well as crevice and stress corrosion. It is exceptionally resistant to chloride-induced pitting. It has high resistance to sulfuric, hydrochloric, acetic acids, chlorine gas, seawater, brines, and many other organic and inorganic chemical solutions that create an aggressive working environment.

**Figure 2:**
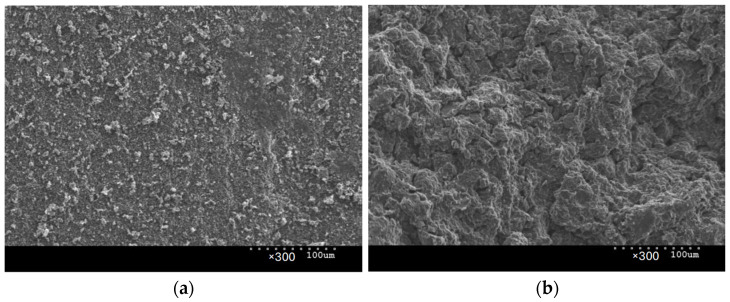
Graphite microstructure observed at a magnification of ×300 in the sample: (**a**) AF-5; (**b**) EDM-180.

Experimental research was conducted on the Charmilles Form 2-LC ZNC machine (GF Solutions, Bienne, Switzerland). The machine is equipped with an isoenergetic transistor generator. The empirical research utilized two POCO graphite electrodes, one with a grain size of 1 µm (AF-5) and the other with a grain size of 10 µm (EDM-180). Based on the analysis of fracture images of the tested electrodes in a scanning electron microscope (Hitachi SU 3500, Hitachi High-Tech, Tokyo, Japan), a clear difference was observed in the grain size of both graphite materials and porosity (Figure 2). The fundamental physical properties of the graphite electrodes from a material data sheet can be found in Table 1 [61]. The graphite electrodes were made of 12 × 12 × 25 mm blocks. The machined workpiece made of Hastelloy C-22 was Ø 10 × 3 mm. Before each experiment, the samples and the tool electrodes were lapped and polished. To eliminate the influence of the variability of the EDM plunge machining conditions along with the change in the depth, it was decided to conduct experiments with the kinematics of free machining, in which the tool electrode worked only with the front surface, and there were no side gaps. The samples and the electrodes were fully immersed in the dielectric based on paraffin hydrocarbons—EDM fluid 108 MP-SE kerosene.

**Figure 3:**
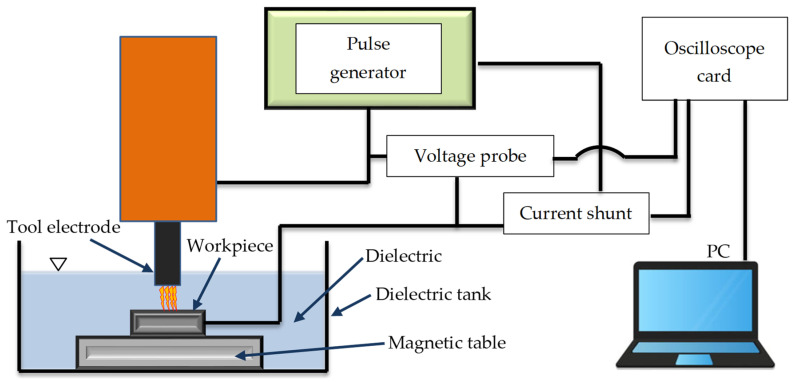
Research set-up plan: voltage and current measurement circuit.

**Figure 4:**
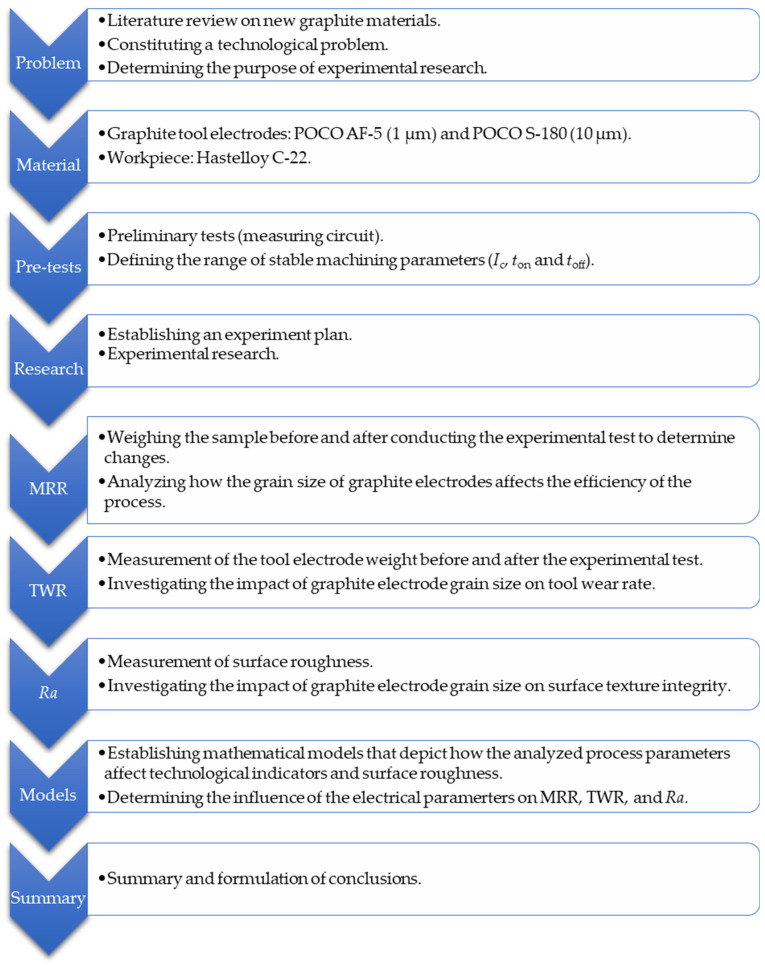
Diagram of the experimental research.

The preliminary examination and review of sources suggest that the effectiveness of material removal is primarily influenced by the current intensity *I*_c_, the electric discharge time *t*_on_, and, to a lesser degree, the time interval between pulses *t*_off_. In the first stage, preliminary tests were carried out to find the range of stable machining parameters, which will be analyzed in the experimental studies. The research stand was configured to a measurement system that identifies the current and voltage waveforms using an NI 5133 oscilloscope card (National Instruments, Austin, TX, USA). Voltage changes were determined using a Tektronix probe. The current intensity values were measured by the voltage drop on a non-inductive current shunt with a constant equivalent resistance value. A research set-up scheme is presented in Figure 3. The signals recorded from both channels on the oscilloscope card were analyzed and processed using the National Instruments DIAdem 2019 software. A diagram illustrating the experimental arrangement is shown in Figure 4.

**Figure 5:**
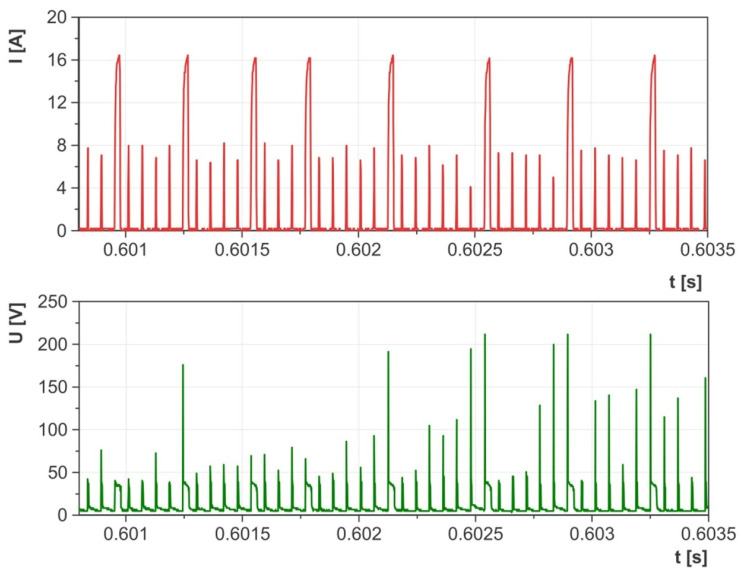
Waveforms of voltage and current measured during EDM for unstable machining conditions.

**Figure 6:**
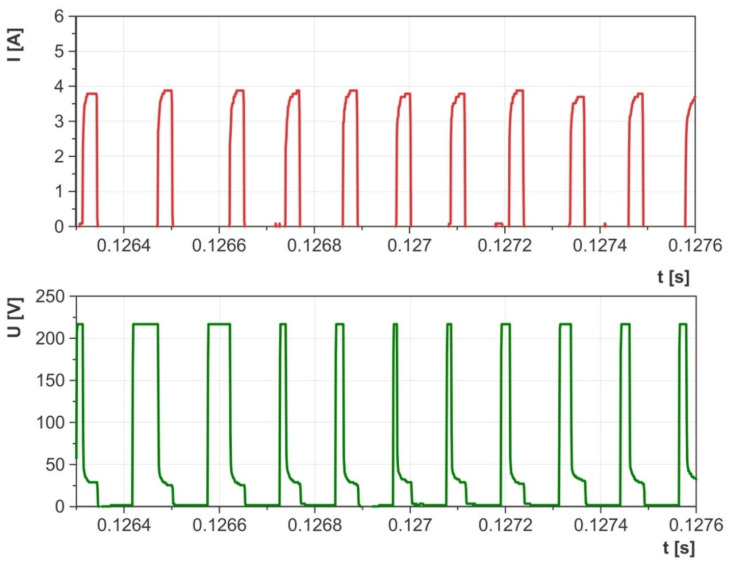
Waveforms of voltage and current measured during EDM for stable machining conditions.

Preliminary tests carried out on the EDM machine with an adapted measuring circuit enabled the range of parameters with stable electrical discharges to be defined. Upon examining the waveforms of the current and voltage, it was noted that at very high current intensities and short discharge times, there were numerous short-circuit impulses that reduced material erosion during EDM with negative polarity (Figure 5). Conversely, at lower current intensities and discharge times, the waveforms of the current and voltage demonstrated high homogeneity and consistency (Figure 6). An equally important parameter that influenced the stability of electrical discharges was the time interval between pulses. Decreasing this interval hinders efficient cleaning of the sparking gap from debris and detached graphite particles, leading to an increased risk of short-circuit impulses and surface damage. The analysis of the waveforms of the discharge current and voltage enabled the selection of stable electrical parameters for the purposes of this study:

- Current intensity *I*_c_ ranging from 1.7 to 5 A;
- Electric discharge time *t*_on_ ranging from 8 to 55 µs;
- The time interval between pulses *t*_off_ ranges from 6 to 75 µs;
- Open voltage: *U*_0_ = 230 V;
- Discharge voltage *U*_c_ = 26 V;
- Tool polarity: negative (workpiece—anode, tool electrode—cathode).

The discharge voltage *U*_c_ and the open voltage *U*_0_ were set at a constant level during the experimental tests. The samples were machined to a depth of 2 mm. A free EDM model was used, in which the tool electrode worked only with its front part, and there were no side gaps. 

**Figure 7:**
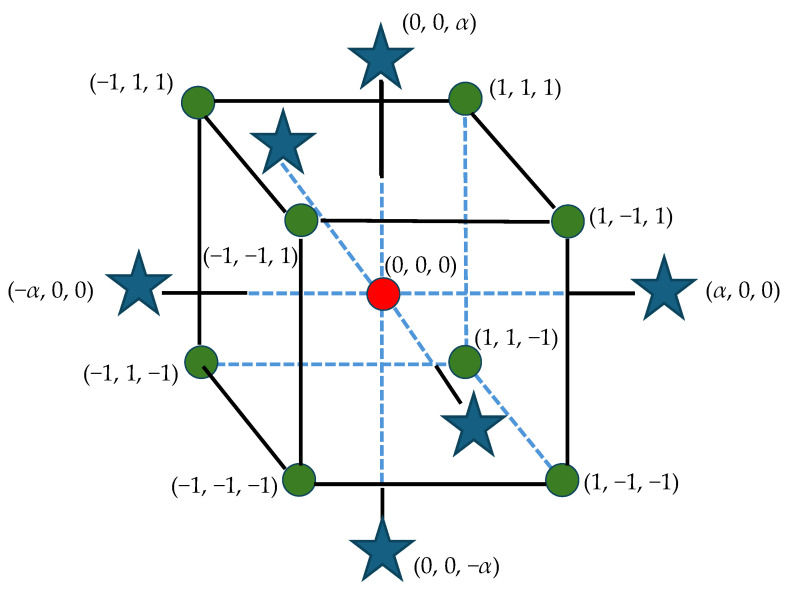
Experimental plan.

Experimental research was carried out to investigate the impact of discharge current *I*_c_, discharge time *t*_on_, and time interval *t*_off_ on tool electrode wear (TWR), material removal rate (MRR), and surface roughness (*Ra*) of Hastelloy C-22 following EDM with negative polarity. Hartley’s experimental design, featuring three input parameters and five levels (Figure 7), was utilized for the study. The levels of machining parameters employed in the experiment are detailed in Table 2. 

For every machined surface, the *Ra* average roughness profile was determined by measuring a 12.5 mm long sampling line consisting of five 2.5 mm elementary sections. The measurements were taken with a Taylor-Hobson FORM TALYSURF Series 2 scan profilometer (Taylor Hobson, Leicester, UK). The measuring device allows measurements with a resolution of 0.6 nm. The rounding radius of the measuring needle was 2 µm, and its opening angle was 45 degrees. Three measurements were taken at various places on each surface, and the *Ra* average value was subsequently calculated. The number of measurement points recorded during a single pass with the measuring needle was 5000 points. The *Ra* parameter, defined in accordance with the PN-EN ISO 4287 standard [62], represents the arithmetic mean of the absolute values of the ordinates *Z*(*x*) within the elementary section *l* (1):

(1) $$Ra=\frac{1}{l}\int_{0}^{l}\left|Z(x)\right|\;dx$$

The rate of tool wear (TWR) and the rate of material removal (MRR) were calculated by analyzing the decrease in weight of both the workpiece and graphite electrode following the EDM process. The weight of the sample was recorded thrice before and after the experiment, and the mean value was calculated. The MRR was obtained by dividing the amount of material removed from the workpiece by the duration of erosion. The proportion of material removed from the tool electrode to the workpiece was utilized to quantify the relative wear of the tool electrode (TWR).

## 3. Results and Discussion

The experimental research focused on exploring the impact of different grain sizes of two POCO graphite electrodes (AF-5 and EDM-180) and electrical variables (such as current intensity *I*_c_, discharge time *t*_on_, and time interval *t*_off_) on performance metrics (MRR and TWR) and surface roughness (*Ra*) of Hastelloy C-22 following EDM with negative polarity. The study was conducted in three phases. The initial phase studied the influence of graphite electrode grain size on process efficiency and tool wear rate. The subsequent phase investigated how the graphite electrodes affected surface roughness (specifically the *Ra* parameter) following machining. Finally, mathematical models were developed to forecast the impact of electrical variables and electrode grain size on TWR, MRR, and *Ra* roughness. The results and analysis for each phase of the study are presented in the subsequent sections.

### 3.1. Material Removal and Tool Wear Rates

The results from the experiments show that the material removal efficiency and relative wear of both graphite tool electrodes vary under the same machining conditions. These results can be found in Table 3. The relative wear of the tool electrode varies in a wide range, and for the AF-5 electrode, it was TWR = 46.19–203.59%. In the case of the EDM-180 electrode, the range was TWR = 50.48–207.59%. In each case, the coarse-grained graphite electrode obtained higher tool wear rates. This happens because graphite has a low effective density and high sublimation temperature, which results in particles being lost during the electro-erosion process. This effect is more pronounced in graphite with larger grain sizes, such as the EDM-180 electrode, which has larger crystals and lower effective density compared to the AF-5 electrode. The main electrical parameter affecting tool wear was the current intensity *I*_c_, followed by the time intervals *t*_off_ and discharge time *t*_on_.

The material removal efficiency of the AF-5 electrode ranged from MRR = 0.95–10.21 mm^3^/min, while for the EDM-180 electrode, it was MRR = 0.84–7.95 mm^3^/min. Using the same parameters for both electrodes, the fine-grained one exhibited better material removal efficiency compared to the coarse-grained graphite electrode. This difference can be attributed to the higher wear rates of the EDM-180 electrode. The increased contamination in the sparking gap from detached graphite grains leads to uneven electrical discharges (resembling Figure 5), ultimately impacting the machining process. This issue was noted during the analysis of the current and voltage waveforms, which showed periodic short-circuit pulses. The AF-5 graphite wears to a lesser extent and is made of smaller grains, which are easier to remove from the sparking gap by the flowing dielectric. Better dielectric renewal provides more intense electrical discharges in the gap.

**Figure 8:**
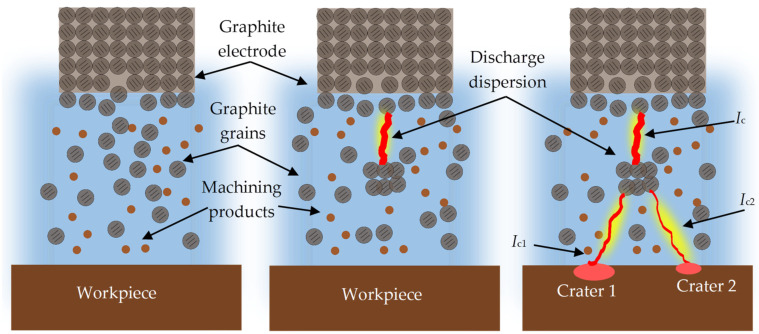
Scheme of electric discharge dispersion in electrical discharge machining.

**Figure 9:**
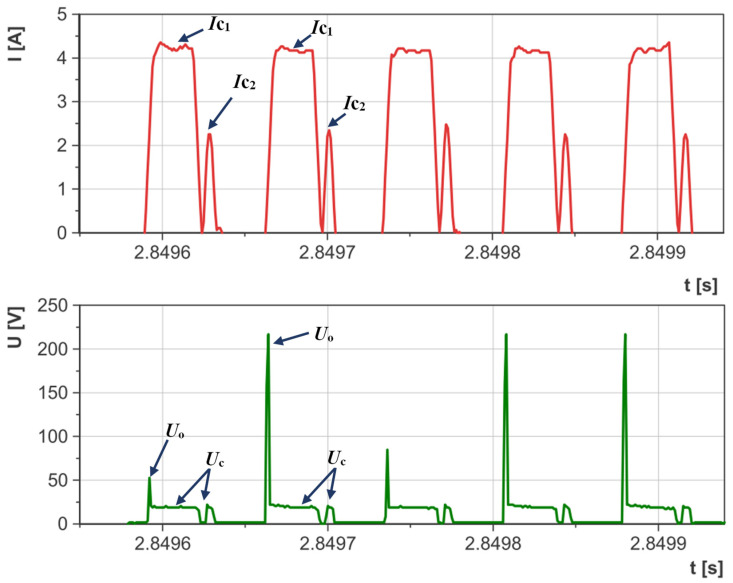
Recorded current and voltage waveform of electric discharge dispersion for the AF-5 electrode and parameter values: *U*_0_ = 230 V, *U*_c_ = 26 V, *I*_c_ = 3.8 A, *t*_on_ = 30 μs, *t*_off_ = 6 μs.

Accumulation of graphite grains in the sparking gap due to electrode wear can change the electric discharge waveform. It was recorded on the example voltage and current waveform (Figure 8 and Figure 9). The dislodged graphite particles move unpredictably between the tool electrode and the workpiece due to the influence of electric field lines. These particles can appear in various places in the form of chains and generate voltage bridges between the anode and the cathode. The effect of bridging the electrodes reduces the voltage in the sparking gap and the dielectric strength of the medium. As a result, the dispersion of the electric discharge on the graphite grains may occur, and the electric discharge with lower energy may be initiated. Similar voltage and current waveforms were observed during powder-mixed electrical discharge machining [63,64,65]. The described phenomenon is random and can occur with an appropriate concentration of graphite grains in the sparking gap. In the case of high wear of the tool electrode and high machining efficiency, excess grains and erosion products between the cathode and the anode increase the probability of a short-circuit pulse. 

### 3.2. Analysis of Surface Integrity

**Figure 10:**
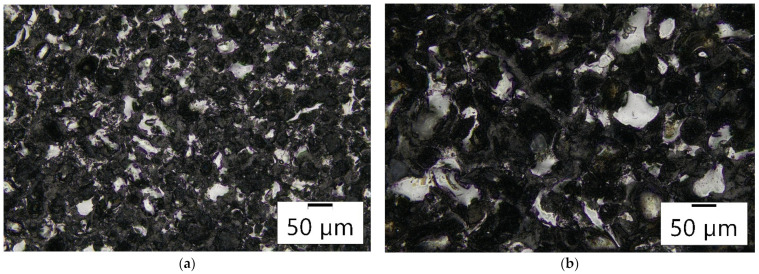
Surface integrity of the workpiece after EDM with the AF-5 electrode for parameters: (**a**) *I*_c_ = 1.7 A, *t*_on_ = 30 µs, *t*_off_ = 37 µs; (**b**) *I*_c_ = 3.8 A, *t*_on_ = 55 µs, *t*_off_ = 37 µs.

**Figure 11:**
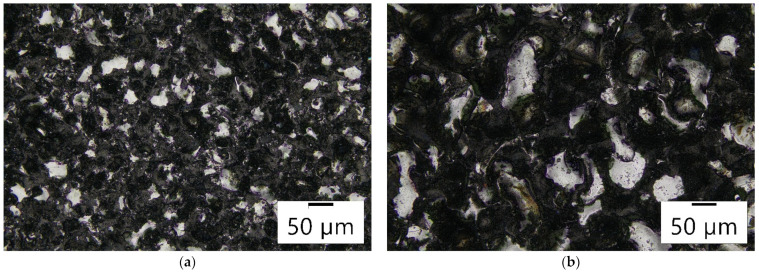
Surface integrity of the workpiece after EDM with the EDM-180 electrode for parameters: (**a**) *I*_c_ = 1.7 A, *t*_on_ = 30 µs, *t*_off_ = 37 µs; (**b**) *I*_c_ = 3.8 A, *t*_on_ = 55 µs, *t*_off_ = 37 µs.

The surface texture following electrical discharge machining is made up of overlapped traces of individual electric discharges in the shape of spherical craters. The surface exhibits an isotropic structure with a high density of local peaks (Figure 10 and Figure 11). The distribution of craters on the machined surface is random. The randomness of the surface texture after the EDM process is linked to the physics of material removal and the varying number of electric discharges on the tool electrode surface. The shape and size of the craters depend on the electrical parameters and physical properties of the graphite electrode. The surface texture after electro-erosion treatment is covered with a black deposit consisting of erosion products, carbon from dielectric pyrolysis, and detached grains of the graphite electrode. Detailed analysis allowed us to observe characteristic microcracks in the surface texture of the workpiece after EDM. The impact of the exceedingly high temperature of the plasma channel melts a particular volume of material. As a result of cooling the melted material by dielectric, tensile stresses are created, which are counteracted by the core material. Microcracks are formed when the ultimate tensile strength is exceeded.

The findings of the experimental study showed that the electrical parameters had a notable impact on the surface roughness (*Ra*). The surface roughness values for the AF-5 electrode varied between 1.25 and 4.4 µm, whereas for the EDM-180 electrode, they ranged from 1.26 to 3.87 µm. In most cases, the surface roughness was comparable or similar for the coarse-grained (EDM-180) and the fine-grained (AF-5) graphite electrode. This proves that the main factor determining the value of *Ra* is the electrical parameters (current intensity *I*_c_, discharge time *t*_on_, and time interval *t*_off_) and, to a lesser extent, the grain size of the graphite electrode. When EDM is conducted with negative polarity, electrons are emitted from the cathode (tool electrode) surface and collide with the workpiece surface. The kinetic energy of the electrons is converted into heat upon collision with the anode, leading to material melting and evaporation from the workpiece. Due to the distribution of thermal energy on the machined surface being the critical factor in negative polarity EDM, the grain size of the graphite electrodes has a negligible impact on surface texture integrity.

### 3.3. Statistical Models of TWR, MRR, and Ra

The research was performed using Hartley’s experimental design approach, involving five levels and three input electrical factors (current intensity *I*_c_, discharge time *t*_on_, and time interval between pulses *t*_off_). To ensure the repeatability of the results, two repetitions were conducted at the central point of the experimental scheme. Following the adopted assumptions of the experiment plan, it consisted of 16 tests with a different set of electrical parameters. Experimental tests were carried out under the same machining conditions for both graphite electrodes (AF-5 and EDM-180).

The experimental results were used to create mathematical models in the form of second-degree regression polynomials that explain how the analyzed process parameters and the graphite electrode grain size affect machining efficiency, tool wear rate, and surface roughness (*Ra*). The regression equations were determined in STATISTICA 13.3 using the step-back regression method.

Each equation has been assigned a correlation coefficient of *r*, a determination coefficient of *R*^2^, and an adjusted determination coefficient of *R*^2^. The *r*-value is an indicator of the strength of the correlation between the input and output data in a regression analysis. A higher *r*-value closer to one indicates a more accurate representation of the variability in the data being studied. To assess the significance of this correlation, the Fisher–Snedecor test (*F*) is used to compare the correlation coefficient obtained. The *F* test value is then compared to the critical value *F*_kr_. A significant *r*-value is considered when the ratio *F*/*F*_kr_ is greater than or equal to 1 for the corresponding *p*-value (*p* = 0.05). Furthermore, the significance of individual terms in the equation is evaluated using Student’s *t*-test. The *t*-test value is calculated and compared to the critical value *t*_kr_ for the associated *p*-value (*p* = 0.05). Terms are considered significant in the equation if the relation *t* ≥ *t*_kr_ holds true. 

Response surface methodology was utilized to construct regression models for the EDM process. The regression equations established for the analyzed technological parameters (MRR and TWR) and surface roughness (*Ra*) exhibit a strong correlation ratio *r*. The *F*/*F*_kr_ ratio exceeds unity significantly in all instances. The standard error of estimate assumes acceptably low values for each regression equation. Table 4 presents a summary of the regression statistics for the specified equations.

**Figure 12:**
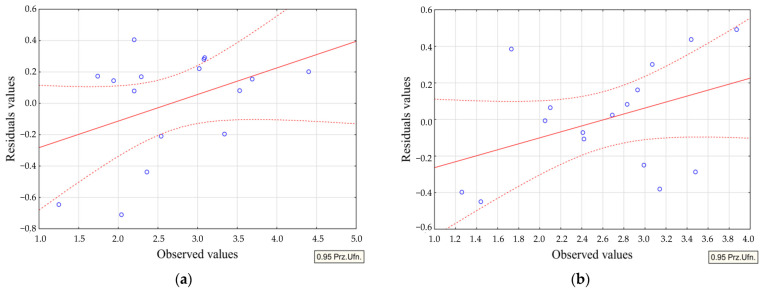
Residuals as a function of observed values for *Ra* models; graphite electrodes: (**a**) AF-5 and (**b**) EDM-180. Red dotted lines—border of the confidence interval; red solid line—regression line; blue circles—residuals values.

For each regression equation, plots of residual values were designated as a function of observed values. Sample plots of residuals for the *Ra* parameter are shown in Figure 12. The analysis allows us to assess the accuracy of fitting experimental data to the determined regression equation. The established mathematical models are characterized by minimal dispersion between the observed and predicted values. It proves the high credibility of the developed mathematical models and the significant impact of the adopted input parameters on the output values tested. 

Following the removal of insignificant variables in the response equations for MRR, TWR, and surface roughness (*Ra*), they were ultimately expressed by a second-order polynomial function.

Equations for machining utilizing the AF-5 graphite electrode (2–4):

MRR = −0.02 − 0.003 × *t_off_^2^* + 0.06 × *I_c_* × *t_off_ +* 0.002 × *t_on_* × *t_off_* (mm^3^/min) (2)

TWR = 429 − 162 × *I_c_* − 0.5 × *t_off_* + 18 × *Ic^2^* (%) (3)

*Ra* = 2.6 − 0.1 × *t_on_* − 0.09 × *I_c_^2^* + 0.04 × *I_c_* × *t_on_* (µm) (4)

Regression equations for the EDM-180 graphite electrode (5–7):

MRR = 0.5 − 0.005 × *t_on_^2^* − 0.001 × *t_off_^2^* + 0.06 × *I_c_* × *t_on_ +* 0.005 × *t_on_* × *t_off_* (mm^3^/min) (5)

TWR = 388 − 143 × *I_c_* + 18 × *I_c_^2^* − 0.2 × *I_c_* × *t_off_* (%) (6)

*Ra* = 1 + 0.5 × *I_c_* − 0.04 × *t_off_* + 0.001 × *t_on_* × *t_off_* (µm) (7)

**Figure 13:**
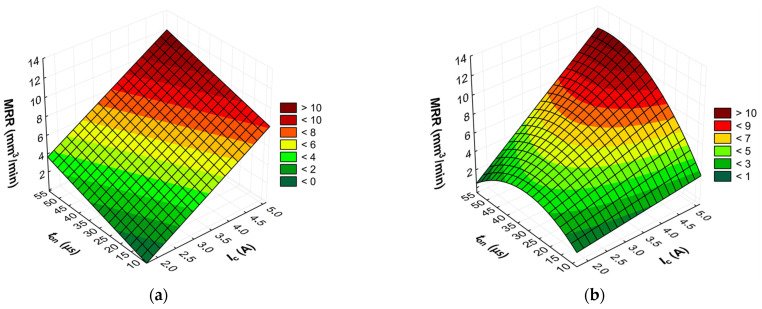
Graphical illustration of the regression equation for MRR (*t*_off_ = 40.5 µs) after EDM with the electrode: (**a**) AF-5 and (**b**) EDM-180.

**Figure 14:**
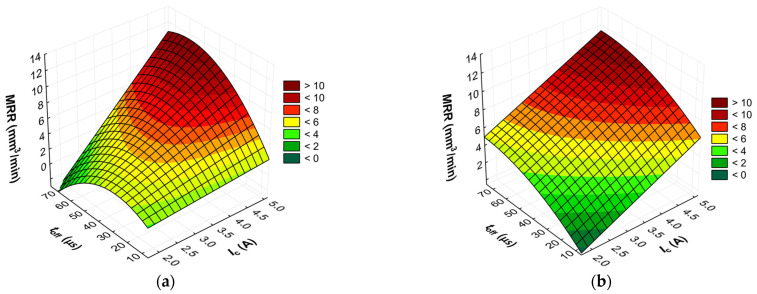
Graphical illustration of the regression equation for MRR (*t*_on_ = 31.5 µs) after EDM with the electrode: (**a**) AF-5 and (**b**) EDM-180.

**Figure 15:**
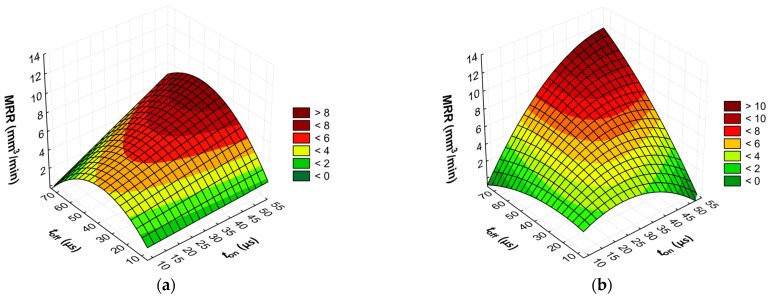
Graphical illustration of the regression equation for MRR (*I*_c_ = 3.4 µs) after EDM with the electrode: (**a**) AF-5 and (**b**) EDM-180.

**Figure 16:**
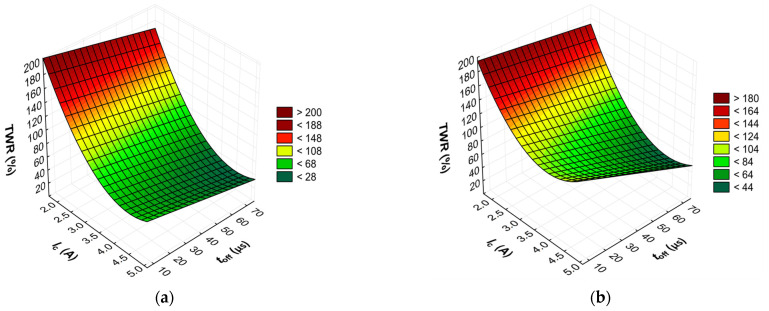
Graphical illustration of the regression equation for TWR of the electrode: (**a**) AF-5 and (**b**) EDM-180.

**Figure 17:**
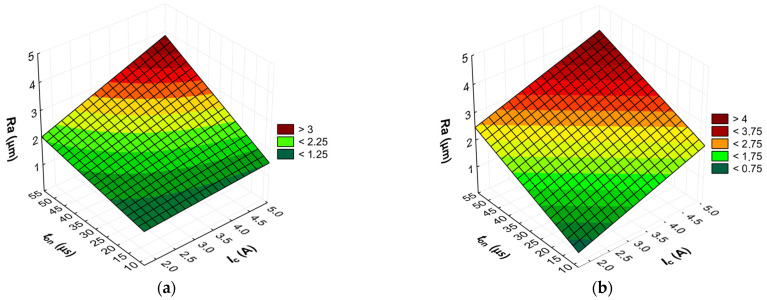
Graphical illustration of the regression equation for *Ra* (*t*_off_ = 40.5 µs) after EDM with the electrode: (**a**) AF-5 and (**b**) EDM-180.

**Figure 18:**
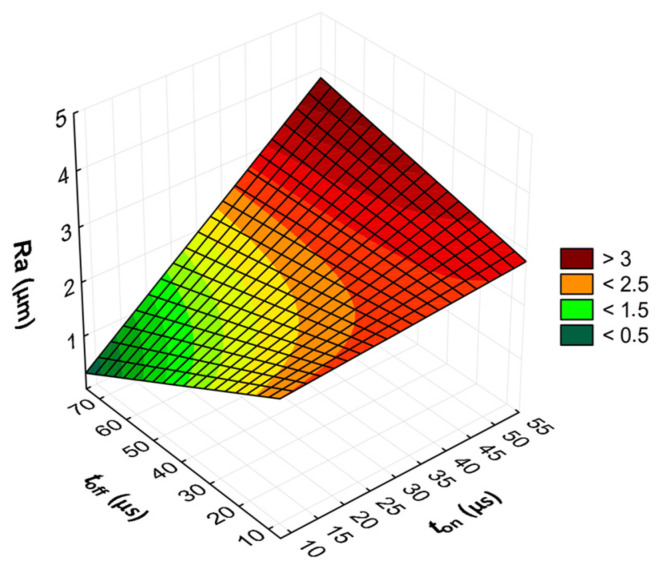
Graphical illustration of the regression equation for *Ra* (*t*_off_ = 40.5 µs) after EDM with the EDM-180 electrode.

The advanced predictive models will allow for the selection of favorable machining conditions based on the desired technological parameters of the EDM process and the surface roughness of the workpiece after machining. The obtained test results subjected to multi-criteria optimization can be generalized and adapted to modern electrical discharge machines. To further comprehend and demonstrate the influence of the EDM process parameters on material removal efficiency, tool electrode wear, and surface roughness, response surface plots were calculated and depicted in Figure 13, Figure 14, Figure 15, Figure 16, Figure 17 and Figure 18. 

Based on the analysis of the regression models developed, it is determined that the effectiveness of material removal depends primarily on the current intensity *I*_c_ and, to a lesser extent, on the discharge time *t*_on_ and the time interval between pulses *t*_off_. Generally, the MRR value increases with the increasing current intensity *I*_c_. This can be attributed to the amount of heat transferred to the material. With an increase in the discharge current *I*_c_, the quantity and velocity of electrons and positively charged ions in the sparking gap also increase. Ions and electrons collide with the surface of the electrodes, leading to a high-temperature increase, which accelerates the melting, evaporation, and sublimation of the workpiece. Additionally, the generated heat speeds up the evaporation of the dielectric liquid and generates high-pressure waves that enhance the removal of molten material from the craters. At lower currents, only a small amount of heat is produced, with some being absorbed by the workpiece, while the remaining heat is absorbed by the dielectric liquid.

**Figure 19:**
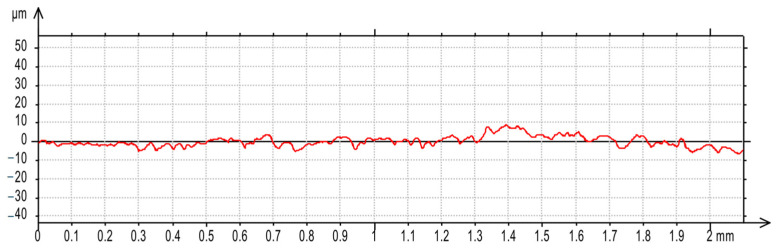
Surface roughness profile graph after EDM with the AF-5 electrode for machining parameters: *I*_c_ = 1.7 A, *t*_on_ = 30 µs, *t*_off_ = 37 µs.

**Figure 20:**
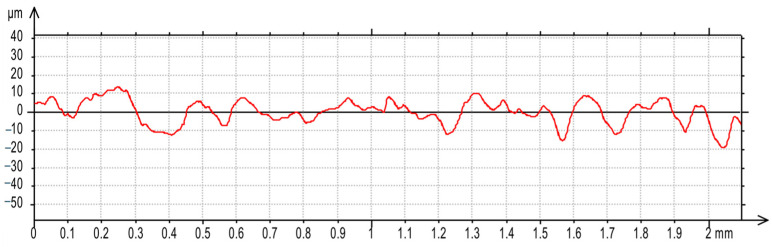
Surface roughness profile graph after EDM with the EDM-180 electrode for machining parameters: *I*_c_ = 3.8 A, *t*_on_ = 55 µs, *t*_off_ = 37 µs.

For the AF-5 graphite electrode, an extension in the discharge time *t*_on_ enhances the effectiveness of material removal due to a more prolonged and intensified heat transfer to the workpiece. Increasing the time interval between pulses *t*_off_ reduces the material removal efficiency due to heat loss and cooling of the heated volume of material by the dielectric liquid. For the EDM-180 graphite electrode, lengthening the time interval *t*_off_ increases process performance. As a result of the high wear of the coarse-grained graphite electrode, the reduction of the time interval between pulses *t*_off_ prevents the removal of machining products and detached large graphite grains from the sparking gap, which increases the probability of short-circuit pulses that reduce process efficiency. Low values of relative tool wear are obtained at high current intensity *I*_c_ and the long-time interval *t*_off_, for both the AF-5 and EDM-180 graphite electrodes. Increased discharge current *I*_c_ heightens the phenomenon of kerosene pyrolysis and carbon deposition on the tool electrode and workpiece surface. The carbon layer creates an additional protective barrier that protects the electrode against wear. Surface roughness is significantly correlated with material removal efficiency. Amplifying the energy of a single electric discharge (discharge time *t*_on_ and current intensity *I*_c_) causes melting and erosion of a sizeable volume of material (Figure 19 and Figure 20). Generated craters are characterized by large diameters and depths, which increases the height parameters of the surface roughness—the *Ra* parameter.

## 4. Conclusions

Experimental studies were focused on determining the influence of graphite electrode grain size (AF-5 and EDM-180) and electrical parameters on the relative tool wear rate (TWR), material removal efficiency (MRR), and surface roughness (*Ra*) of Hastelloy C-22 after EDM with negative polarity. The impact of grain size and process parameters on TWR, MRR, and *Ra* parameters was analyzed and determined. A study on the material removal mechanism using graphite electrodes of varying grain sizes was carried out, with particular emphasis on the wear mechanism of graphite electrodes. Observations and analysis of the surface texture integrity after machining were performed, and surface roughness was measured—the *Ra* parameter. In the final section, mathematical equations were developed to demonstrate how the analyzed process parameters affect technological indicators and surface roughness. Conclusions were drawn based on the experimental results obtained:

1. Material removal efficiency is mainly determined by the contamination of the sparking gap by detached grains of the graphite electrode and their impact on the electric discharge waveform.
2. Fine-grained graphite electrodes are recommended during electrical discharge machining with negative polarity due to lower tool wear rate and easier removal of the detached small graphite grains from the sparking gap.
3. Detached graphite grains can influence the change of current intensity and discharge voltage in the sparking gap. With an appropriate concentration of graphite grains in the gap, the phenomenon of electric discharge dispersion may occur. When the sparking gap is heavily contaminated with machining debris and large graphite particles, the probability of short-circuit pulses occurring is heightened.
4. The size of the grain in the graphite electrodes has little to no impact on the surface roughness (*Ra*).
5. The wear rate of the tool electrode was reduced when using the AF-5 graphite because of its finer grain size and lower effective density.
6. The main electrical parameter determining the relative tool wear of the graphite electrode was the discharge current *I*_c_.
7. Material removal efficiency significantly depends on the current intensity *I*_c_, the electrical discharge time *t*_on_, and, to a lesser extent, the time interval between the pulses *t*_off_.
8. An elevation in the energy of an individual electric discharge increases the surface roughness, as indicated by the *Ra* parameter.

## Figures and Tables

**Table 1:** Physical properties of POCO graphite electrodes [61].

POCO Graphite	Average Grain Size (µm)	Electrical Resistivity (µΩm)	Effective Density (g/cm^3^)	Flexural Strength (MPa)	Shore Hardness
AF-5	1	21.6	1.80	117	87
EDM-180	10	13.0	1.78	58	66

**Table 2:** Scheme of design for process parameters and their respective levels.

Level	EDM Parameters		
	Discharge Time *t*_on_ (µm)	Time Interval *t*_off_ (µm)	Discharge Current *I*_c_ (A)
−1.68	8	6	1.7
−1	17	19	2.7
0	30	37	3.8
1	41	51	4
1.68	55	75	5

**Table 3:** Results of experimental studies.

Ex. No.	EDM Parameters			AF-5			EDM-180		
	*I*_c_ (A)	*t*_on_ (µs)	*t*_off_ (µs)	MRR (mm^3^/min)	TWR (%)	*Ra* (µm)	MRR (mm^3^/min)	TWR (%)	*Ra* (µm)
1	2.7	17	19	2.60	116.38	2.20	3.02	84.03	2.10
2	2.7	17	51	2.04	77.41	1.94	1.82	81.47	1.73
3	2.7	41	19	2.84	103.60	2.04	2.01	120.20	2.42
4	2.7	41	51	3.76	81.73	2.54	3.30	86.03	2.69
5	4	17	19	3.43	59.97	2.20	3.17	150.00	2.83
6	4	17	51	4.83	50.58	2.29	4.34	54.12	2.05
7	4	41	19	3.96	69.31	3.69	2.33	90.41	2.99
8	4	41	51	9.66	49.17	3.34	8.40	50.48	3.87
9	1.7	30	37	0.95	203.59	1.25	0.84	207.59	1.26
10	5.0	30	37	10.21	46.19	3.53	7.95	51.99	3.14
11	3.8	8	37	2.59	61.82	1.74	2.30	66.42	1.44
12	3.8	55	37	4.92	68.01	4.40	4.91	71.13	3.48
13	3.8	30	6	2.75	78.18	3.08	3.26	79.05	3.44
14	3.8	30	75	5.18	51.96	2.36	4.71	55.19	2.41
15	3.8	30	37	7.52	54.94	3.02	6.03	58.30	2.93
16	3.8	30	37	7.97	53.97	3.09	6.49	56.18	3.07

**Table 4:** Regression summary.

POCO Graphite	Investigated Parameters	Calculated Regression Statistics				
		Ratio *r*	Ratio *R*^2^	Adjusted *R*^2^	Standard Error of Estimation	*F*/*F*_kr_
AF-5	MRR	0.96	0.93	0.90	0.87	2.2
	TWR	0.95	0.91	0.89	13.08	5.8
	*Ra*	0.91	0.83	0.82	0.35	3.6
EDM-180	MRR	0.92	0.84	0.79	1.02	2.5
	TWR	0.82	0.66	0.54	28.67	1.1
	*Ra*	0.92	0.84	0.81	0.33	3.6

## Data Availability

The data presented in this study are available on request from the corresponding author. The data are not publicly available due to privacy.
